# Validation of a prediction model for long-term outcome of aphasia after stroke

**DOI:** 10.1186/s12883-018-1174-5

**Published:** 2018-10-15

**Authors:** Femke Nouwens, Evy G. Visch-Brink, Hanane El Hachioui, Hester F. Lingsma, Mieke W. M. E. van de Sandt-Koenderman, Diederik W. J. Dippel, Peter J. Koudstaal, Lonneke M. L. de Lau

**Affiliations:** 1 Department of Neurology, Erasmus MC University Medical Center, room Nb-324, PO Box 2040, 3000 CA Rotterdam, the Netherlands; 2 Rijndam Rehabilitation, Rotterdam, the Netherlands; 3 Department of Public Health, Erasmus MC University Medical Center, Rotterdam, the Netherlands; 4 Department of Rehabilitation Medicine, Erasmus MC University Medical Center, Rotterdam, the Netherlands; 5 Slotervaart Medical Center, Department of Neurology, Amsterdam, the Netherlands

**Keywords:** Aphasia, Stroke, Prognostic model, Validation, Outcome

## Abstract

**Background:**

About 30% of stroke patients suffer from aphasia. As aphasia strongly affects daily life, most patients request a prediction of outcome of their language function. Prognostic models provide predictions of outcome, but external validation is essential before models can be used in clinical practice. We aim to externally validate the prognostic model from the Sequential Prognostic Evaluation of Aphasia after stroKe (SPEAK-model) for predicting the long-term outcome of aphasia caused by stroke.

**Methods:**

We used data from the Rotterdam Aphasia Therapy Study – 3 (RATS-3), a multicenter RCT with inclusion criteria similar to SPEAK, an observational prospective study. Baseline assessment in SPEAK was four days after stroke and in RATS-3 eight days. Outcome of the SPEAK-model was the Aphasia Severity Rating Scale (ASRS) at 1 year, dichotomized into good (ASRS-score of 4 or 5) and poor outcome (ASRS-score < 4). In RATS-3, ASRS-scores at one year were not available, but we could use six month ASRS-scores as outcome. Model performance was assessed with calibration and discrimination.

**Results:**

We included 131 stroke patients with first-ever aphasia. At six months, 86 of 124 (68%) had a good outcome, whereas the model predicted 88%. Discrimination of the model was good with an area under the receiver operation characteristic curve of 0.87 (95%CI: 0.81–0.94), but calibration was unsatisfactory. The model overestimated the probability of good outcome (calibration-in-the-large *α* = − 1.98) and the effect of the predictors was weaker in the validation data than in the derivation data (calibration slope *β* = 0.88). We therefore recalibrated the model to predict good outcome at six months.

**Conclusion:**

The original model, renamed SPEAK-12, has good discriminative properties, but needs further external validation. After additional external validation, the updated SPEAK-model, SPEAK-6, may be used in daily practice to discriminate between patients with good and patients with poor outcome of aphasia at six months after stroke.

**Trial registration:**

RATS-3 was registered on January 13th 2012 in the Netherlands Trial Register: NTR3271. SPEAK was not listed in a trial registry.

**Electronic supplementary material:**

The online version of this article (10.1186/s12883-018-1174-5) contains supplementary material, which is available to authorized users.

## Background

Aphasia occurs in approximately 30% of stroke patients and has a strong impact on everyday communication and daily functioning [1, 2]. Shortly after stroke, patients and their family are faced with major uncertainties regarding recovery of communication. Consequently, there is a need for individual estimation of the expected outcome. Adequate personal prognosis may also contribute to optimizing individual care, which is important as medical and paramedical care becomes increasingly personalized [3]. Furthermore, predictions of outcome may corroborate rationing of care, in order to better distribute limited resources. Prediction of post-stroke aphasia outcome is often based on models that consist of determinants identified in a single dataset, e.g. age, sex, aphasia severity and subtype; site, size and type of the lesion; vascular risk factors and stroke severity [4–11]. Before a model can be used in daily practice, it should be externally validated [3, 12]. This means that the generalizability of a model is assessed in different cohorts with more recent recruitment (temporal validation), from other institutions (geographical validation), and by different researchers [3]. To our knowledge, none of the few available prognostic models predicting language outcome has been externally validated [13–16].

Previously, our group has constructed a prognostic model for the outcome of aphasia due to stroke. The model was derived from the dataset of the Sequential Prognostic Evaluation of Aphasia after stroKe (SPEAK) study, and performed well [13]. The aim of the current study was to externally validate the SPEAK-model in an independent, yet comparable cohort of stroke patients with aphasia.

## Methods

### The SPEAK-model

SPEAK was an observational prospective study in 147 patients with aphasia due to stroke conducted between 2007 and 2009 in the Netherlands [13]. Demographic, stroke-related and linguistic characteristics of 130 participants, collected within six days of stroke, were used to construct a model predicting good aphasia outcome one year after stroke, defined by a score of 4 or 5 on the Aphasia Severity Rating Scale (ASRS) from the Boston Diagnostic Aphasia Examination [17]. This scale is used for rating communicative ability in spontaneous speech. The ScreeLing, an aphasia screening test designed to assess the core linguistic components semantics, phonology and syntax in the acute phase after onset, was also included in the model [18–20]. For detailed methods, results and discussion we refer to the original paper [13]. The final SPEAK-model contained six baseline variables: ScreeLing Phonology score, Barthel Index score, age, level of education (high/low), infarction with a cardio-embolic source (yes/no) and intracerebral hemorrhage (yes/no) (Online Additional file 1, Box A1). This model explained 55.7% of the variance in the dataset. Internal validity of the model was good, with an AUC (Area Under the Curve, where the curve is the Receiver Operation Characteristic (ROC) curve) of 0.89 [13].

### Validation

For external validation of the SPEAK-model we used data from the Rotterdam Aphasia Therapy Study (RATS) – 3, a randomized controlled trial (RCT) studying the efficacy of early initiated intensive cognitive-linguistic treatment for aphasia due to stroke, conducted between 2012 and 2014 [21, 22]. RATS-3 was approved by an independent medical ethical review board. Details about the study design, methods and results have been reported elsewhere and a summary will be provided below [21, 22].

### Participants and recruitment

A total of 23 hospitals and 66 neurorehabilitation institutions across the Netherlands participated in RATS-3. The majority of participating institutions and local investigators (90%) differed from those involved in SPEAK. In- and exclusion criteria for both studies are presented in Table 1.

**Table 1:** In- and exclusion criteria for participants in RATS-3 and in the SPEAK cohort

	RATS-3	SPEAK
Inclusion:	First-ever aphasia due to stroke	First-ever aphasia due to stroke
	Aphasia ascertained by a speech and language therapist using the 36-item Token Test^a^ and/or a score < 5 on the ASRS	Aphasia ascertained by a neurologist and a speech and language therapist
	Testable with the ScreeLing	A score below the cut-off point of the Token Test and/or the ScreeLing
	Within two weeks of stroke onset	Within two to six days of stroke onset
	Age between 18 and 85	Adult
	Language near-native Dutch	Language near-native Dutch
	A life expectancy of >six months	
	Able to tolerate intensive treatment	
Exclusion:	A subarachnoid or subdural hemorrhage	
	Success or feasibility of intensive language treatment was severely threatened by:	Presence of one of the following criteria:
	- severe dysarthria	- severe dysarthria
	- premorbid dementia	- pre-stroke dementia (suspected or confirmed)
	- illiteracy	- illiteracy
	- severe developmental dyslexia	- developmental dyslexia
	- severe visual perceptual disorders	- severe perceptual disorders of vision or hearing
	- recent psychiatric history	- psychiatric history

^a^De Renzi, E, Faglioni, P. Normative data and screening power of a shortened version of the Token Test. Cortex 1978;14:41–49

### Prognostic variables

Patients with aphasia due to stroke were included in RATS-3 within 2 weeks of stroke. At inclusion, the following baseline variables were recorded: age, sex, education level, stroke type (cerebral infarction or intracerebral hemorrhage), ischemic stroke subtype (with or without a cardio-embolic source). Level of independence was estimated with the Barthel Index, a questionnaire containing ten items about activities of daily life [23]. All participants were tested with the ScreeLing to detect potential deficits in the basic linguistic components [19, 24]. Spontaneous speech samples were collected with semi-standardized interviews according to the Aachen Aphasia Test-manual [25]. Aphasia severity was assessed by scoring the spontaneous speech samples with the ASRS.

### Outcome

In SPEAK, ASRS-scores were used to assess aphasia outcome [17]. This six point scale is used to rate spontaneous speech and ranges from 0: “No usable speech or auditory comprehension” to 5: “Minimal discernible speech handicaps; the patient may have subjective difficulties which are not apparent to the listener”. The SPEAK-model predicts the occurrence of ‘good outcome’, i.e. an ASRS-score of 4 or 5 after 1 year. In RATS-3 follow-up was at 4 weeks, 3 and 6 months after randomization. ASRS-scores from the RATS-3 cohort at 6 months after randomization were used as outcome in the analysis, as this was closest in time to the original model.

### Statistical analyses

Outcome in the RATS-3 cohort was divided in good (ASRS-score of 4 or 5) or poor (ASRS-score < 4). To validate the SPEAK-model we assessed discrimination and calibration [3, 12, 26–28]. For both analyses predicted probability of a good outcome was calculated using the SPEAK-model (Online Additional file 1, Box A1).

Discriminative properties of the model were summarized with the *c* index, similar to the AUC. Good discrimination means that the model is able to reliably distinguish patients with good aphasia outcome from those with poor outcome.

We assessed the calibration properties of the model by studying to what extent the predicted probability of aphasia outcome corresponded with the observed outcome. A calibration plot was constructed by ordering the predicted probabilities of good aphasia outcome ascendingly and forming five equally large groups. Per group, the mean probability of a good outcome at 6 months was calculated, resulting in five predicted risk-groups. Subsequently, in each risk-group, proportions were calculated of participants with an observed good outcome. These proportions were plotted against the mean probability of a good outcome predicted by the SPEAK-model. Outcomes of the linear predictor *y*, calculated with the SPEAK-model, were used to fit a logistic regression model predicting the dichotomous outcome of good versus poor outcome to assess calibration-in-the-large and the calibration slope. If calibration of a model is optimal, the calibration-in-the-large *α* equals 0 and the calibration slope *β* equals 1. In case of insufficient calibration we will recalibrate the prognostic model by adjusting the intercept.

### Handling of missing data

For participants with missing outcome scores at 6 months, scores at 3 months after randomization were used. If no scores were available at 3 months, patients were excluded. Missing data for the other variables were imputed using simple imputation: for binary and categorical variables the mode was imputed and means were used for continuous variables.

## Results

No outcome data at 6 months were available in 28 of 153 participants, and one participant was excluded because aphasia was later found to be caused by a brain tumor. Reasons for missing outcome data were death (*n* = 7), serious illness (*n* = 4), refusal (*n* = 16) and emigration abroad (n = 1). Of these 28 patients, 21 participants were excluded because outcome at 3 months was also not available. For 7 participants we used ASRS-scores at 3 months to impute missing values at 6 months. Baseline data of patients in the validation sample (*n* = 131), as well as those from the SPEAK cohort (*n* = 147) are provided in Table 2. Groups differed slightly with respect to the baseline variables sex, level of education, type of stroke and aphasia severity.

**Table 2:** Baseline model parameters of participants in the original SPEAK cohort and in RATS-3

	SPEAK cohort (*n*= 147) Derivation cohort	RATS-3 cohort (*n*= 131) Validation cohort
Age, mean (SD), in years	67 (15)	65 (12)
Sex, n (%female)	78 (53%)	56 (43%)
Level of education, n (%)		
High ^■^	55 (42%)	60 (46%)
Low^▲^	74 (57%)	71 (54%)
Unknown ♦	2 (2%)	0
Type of stroke, n (%)		
Non-cardio-embolic infarction	84 (57%)	81 (62%)
Cardio-embolic infarction	42 (29%)	23 (18%)
Intracerebral hemorrhage	21 (14%)	24 (18%)
Unknown ♦	0	3 (2%)
Time since onset to inclusion, mean (range), in days	4 (2–6)	8 (1–18)
Barthel Index, median (IQR) ♦	15 (7.75–20)	16 (6–20)
ScreeLing Phonology score, mean (SD)○	14 (6)	15 (6.5)
ASRS-scores at baseline, n (%)		
Score 0	18 (12%)	17 (13%)
Score 1	28 (19%)	21 (16%)
Score 2	33 (22%)	28 (21%)
Score 3	26 (18%)	38 (29%)
Score 4	27 (18%)	27 (21%)
Score 5	3 (2%)	0
Missing	12 (8%)	0

^■^ High = senior vocational education, higher education or university

^▲^ Low = no/unfinished elementary school, elementary school, unfinished junior secondary vocational education or junior secondary vocational education

♦ Imputed scores used for analysis: level of education = low; type of stroke = non-cardio-embolic infarction; Barthel Index score = 13 (*n* = 14)

○ ScreeLing Phonology scores range from 0 to 24

**Fig. 1:**
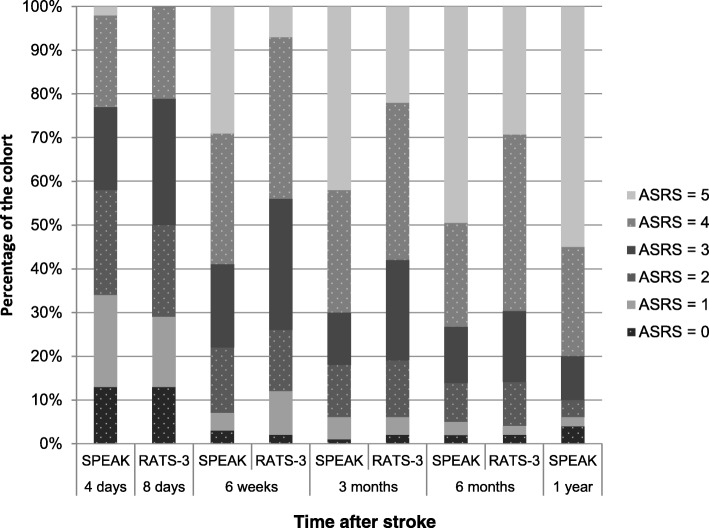
ASRS-scores over time in SPEAK and RATS-3. ASRS-scores: 5 = minimal discernible speech handicap, some subjective difficulties that are not obvious to the listener; 4 = some obvious loss of fluency in speech or facility of comprehension, without significant limitation in ideas expressed or form of expression; 3 = able to discuss almost all everyday problems with little or no assistance, reduction of speech and/or comprehension; 2 = conversation about familiar topics is possible with help from the listener, there are frequent failures to convey an idea; 1 = all communication is through fragmentary expression, great need for inference, questioning and guessing by listener, limited information may be conveyed; 0 = no usable speech or auditory comprehension

In the derivation SPEAK cohort (*n* = 130), 11% of the patients had an ASRS-score of 4 or 5 at baseline (4 days after stroke) and 78% had a good outcome after 1 year. In the RATS-3 cohort we found a proportion of 21% with a score of 4 or 5 at baseline (8 days after inclusion) and 68% at 6 months. This is comparable to the 74% in SPEAK at six months. The course of ASRS-scores in the RATS-3 and SPEAK cohort over time is presented in Fig. 1.

**Fig. 2:**
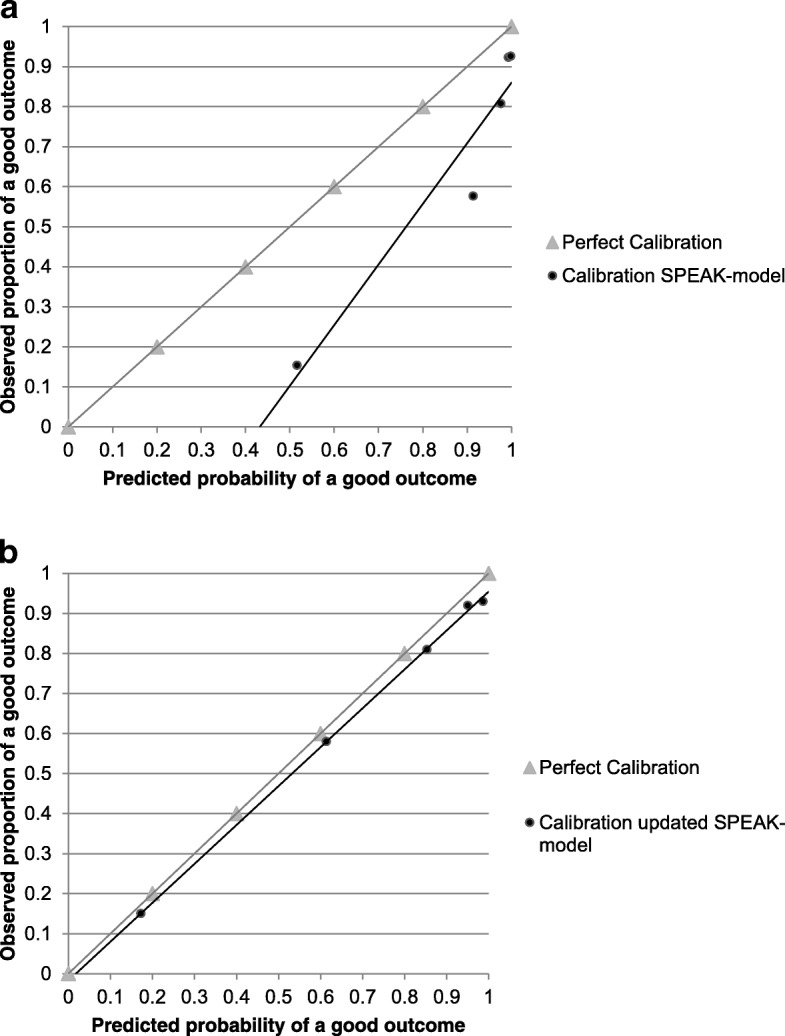
Calibration plots of the SPEAK-model and updated SPEAK-model. **a** Calibration plot of the original SPEAK-model, SPEAK-12. **b** Calibration plot of the updated SPEAK-model, SPEAK-6

Discrimination of the SPEAK-model was good, with an AUC of 0.87 (95% confidence interval: 0.81 to 0.94). In Fig. 2a, the grey line depicts calibration of a hypothetically perfect model and the 5 dots represent calibration values in the five subgroups of patients, ordered by increasing predicted probabilities and plotted against the actual proportions of good outcome. The mean predicted probability of good aphasia outcome at 1 year was 88%, while the observed percentage was 68%, but this was measured at 6 months. The SPEAK-model was too optimistic in predicting good aphasia outcome, with calibration-in-the-large of *α* = − 1.98. The calibration slope of *β* = 0.88 indicated that the predictor effects were slightly weaker in the validation data than in the derivation data.

As Fig. 1 shows that there is still improvement after 6 months, we assume that the poor calibration-in-the-large is at least partly due to the different timing of the outcome measurement; 6 months versus 1 year. Thus, we updated the SPEAK-model to predict outcome at 6 months instead of 1 year by adapting the intercept (Online Additional file 1, Box A2). After revising the SPEAK-model, the calibration slope remained *β* = 0.88, but calibration-in-the-large improved considerably: *α* = − 0.24 (Fig. 2b). We suggest renaming the original SPEAK-model predicting outcome at 1 year after stroke into SPEAK-12 and naming the updated model SPEAK-6.

## Discussion

We aimed to externally validate the published SPEAK-model for the long-term prognosis of aphasia due to stroke using data from an independent cohort of stroke patients with aphasia, RATS-3. The SPEAK-model performed very well in terms of discriminating between good (ASRS 4 or 5) and poor (ASRS < 4) outcome. However, calibration was suboptimal, as it was overoptimistic in predicting good aphasia outcome, partly due to the difference in timing of the outcome which was 1 year in SPEAK and 6 months in RATS-3. Therefore, we proposed an updated version of the SPEAK-model for the prediction of outcome at 6 months.

Prognostic models are used in clinical practice to predict possible outcomes or risks of acquiring certain diseases. To our knowledge, apart from the SPEAK-model, only three other models to predict outcome of aphasia after stroke have been published [14–16]. One logistic regression model predicting early clinical improvement in stroke patients with aphasia was constructed based on findings from CT-angiography and CT-perfusion [15]. Clinical applicability of this model is limited, as these detailed CT-data are rarely available in daily practice. Another logistic regression model addressed the effect of speech and language treatment (SLT) on communication outcomes [14]. The authors found that the amount of SLT, added to baseline aphasia severity and baseline stroke disability significantly affected communication 4 to 5 weeks after stroke. Baseline variables were recorded within 2 weeks of stroke. Recently, a model was published predicting everyday communication ability (Amsterdam-Nijmegen Everyday Language Test; ANELT) at discharge from inpatient rehabilitation based on ScreeLing Phonology and ANELT-scores at rehabilitation admission [16]. These models predict outcome of aphasia recovery only in patients treated with SLT, but do not predict outcome before treatment is initiated. Furthermore, in both studies the cohort included only patients eligible for intensive treatment.

For a prognostic model to be valid and reliable, it is important to evaluate the clinical applicability and generalizability of the model [29]. Inclusion criteria in SPEAK and RATS-3 were not strict, so that both cohorts can be considered representative of acute stroke patients with aphasia in general. The SPEAK-model is valuable for predicting aphasia outcome early after stroke in clinical practice as it includes easily available baseline variables [13]. It requires only the Barthel Index score and the ScreeLing Phonology score to be collected outside clinical routine. The Barthel Index is commonly assessed in the acute phase, allowing for application of this model without much effort [30].

Our study is the first to validate a model for the prognosis of aphasia outcome in an independent cohort. Determining whether a model generalizes well to patients other than those in the derivation cohort, is crucial for the application of that model in daily practice [12, 26, 27, 29]. We found that the SPEAK-model is able to adequately distinguish stroke patients with aphasia who will recover well with respect to functional verbal communication from patients who will not. The model appears less accurate when it comes to the comparison of predicted and actual good outcome.

A first possible explanation may be the different intervention in the two studies. In SPEAK, patients received usual care and researchers did not interfere with the treatment provided. In RATS-3, treatment was strictly regulated, as in this RCT patients were randomly allocated to 4 weeks of either intensive cognitive-linguistic treatment or no treatment, starting within 2 weeks after stroke. After this period both groups received usual care, as in SPEAK. In RATS-3 we found no effect of this early intervention and both intervention groups scored equally on all outcomes. Thus, we believe treatment does not explain the poor calibration.

Second, there was a difference between SPEAK and RATS-3 with respect to the interval between stroke onset and inclusion of patients. In SPEAK, patients were included on average 4 days after onset and in RATS-3 after 8 days. This seemingly small difference might in fact have caused substantial differences in the prognostic effect of the baseline ScreeLing and Barthel Index scores. Recovery can occur rapidly early after stroke, as was shown in the SPEAK cohort, with a statistically significant improvement on the ScreeLing Phonology score between the first and second week after stroke [31]. Hence, these predictors might have different effects in the RATS-3 cohort, as represented in the suboptimal calibration slope.

Third and most importantly, calibration is likely to have been influenced by a different follow up duration, which was 6 months in RATS-3 versus 1 year in SPEAK. In SPEAK, ASRS-scores improved significantly up to 6 months after aphasia onset, but no significant improvement was found between 6 and 12 months [31]. We used this finding for the design of the present study to justify the earlier time-point for the outcome in RATS-3. Although in SPEAK no statistically significant improvement in ASRS-scores was found between 6 and 12 months after stroke, some improvement still occurred [31]. Of the participants from SPEAK 74% had an ASRS-score of 4 or 5 at 6 months after stroke, which is fairly similar in RATS-3 at that time-point (68%). It is likely that calibration would have been better if the outcome was determined at 12 months in the RATS-3 cohort, because of the small, but apparent recovery between 6 and 12 months after stroke.

We therefore suggest an updated version, SPEAK-6, to predict outcome at 6 months. More extensive updating could imply refitting the models to the new dataset, to obtain new model coefficients [32–34]. However, as the model discrimination was good, we updated only the intercept to make the model applicable to predict outcome at 6 months, when the average probability of a good outcome is lower than at 1 year. We recommend that the updated SPEAK-6 is validated in the future in new independent datasets.

This study shows again that the external validity of prognostic models in new settings should always be carefully assessed. However, it should also be noticed that perfect calibration might in fact be impossible, as it implies that a model perfectly predicts outcome for all patients [35].

### Strengths and limitations

The major limitation of this validation study is the difference in time post onset at which predictor and outcome data were collected. Strength is that the RATS-3 and SPEAK cohorts are comparable, due to similar inclusion criteria. However, whereas participation in SPEAK merely involved periodic language evaluations, RATS-3 was an intervention trial, with either early intensive treatment or no early treatment. Due to these experimental interventions many patients refused participation. Also, selection criteria for RATS-3 were slightly stricter than in SPEAK regarding the potential to receive early intensive treatment. Consequently, the SPEAK and RATS-3 cohorts might represent slightly different populations of stroke patients with aphasia, albeit closely related [26]. Therefore, as in all clinical trials, one must be careful in generalizing the results to all stroke patients with aphasia [36].

Although both the derivation cohort and the validation cohort consist of well over a hundred participants, sample sizes may be considered rather small for adequate modelling [28, 36]. This is reflected in the slight imbalance of baseline characteristics between both study cohorts. This imbalance may underpin the necessity of larger sample sizes to better reflect the population of stroke patients with aphasia. Furthermore, in both cohorts a fairly large proportion of patients with mild aphasia (baseline ASRS > 3) was included. Although this was a reflection of the population, it may have influenced the model, as patients with mild aphasia are known to often fully recover [6, 37].

A much debated issue is the potential lack of sensitivity of rating scales for analyses of spontaneous speech in aphasia [38]. In the current study, we dichotomized outcome, further reducing sensitivity. It can be argued that the definition of “good outcome” with an ASRS of 4 or 5 is somewhat optimistic. A score of 4, or sometimes even 5, does not imply full recovery. Patients with a score of 4 still experience difficulties with word finding or formulating thoughts into language.

The ScreeLing is currently only available in Dutch, which severely limits the applicability of the prediction model. However, adaptation to other languages should not be very complicated as the ScreeLing Phonology subscale contains well-known tasks to measure phonological processing, e.g. repetition, discrimination of minimal pairs, and phoneme/grapheme conversion [20]. Including linguistic functioning, as a possible predictor in prognostic models seems essential, as linguistic functioning, in particular phonology, appears to be a better predictor than overall aphasia severity [13, 16, 39]. At the moment the ScreeLing is being translated into English, German and Spanish, and more languages are to come.

The Barthel Index was included in the original model as a measure of overall stroke severity. Although its reliability is good, it may be debated whether the Barthel Index score accurately reflects stroke severity, as the score is influenced by factors related to health care management choices, such as receiving a urinary catheter or feeding tube on intensive care units. Despite its shortcomings the Barthel Index was found to be a valid outcome measure for stroke trials [40].

Finally, the RATS-3 database contained several missing values. Of the participants who refused evaluation at 6 months, three had fully recovered, which may have introduced a slight bias. Missing values for other variables in the model mostly resulted from inconsistencies in reporting the scores. We used generally accepted methods for imputation of the data and for most variables few data were missing (< 5%) [27]. For the Barthel Index 10% had to be imputed, which is a fairly large proportion. There were no clear reasons for these missing values, other than clinicians sometimes just forgot to fill out the score form, which in our view justifies imputation.

## Conclusion

The original SPEAK-model, renamed SPEAK-12, performs well in predicting language outcome after 1 year in patients with aphasia due to stroke. As calibration was initially unsatisfactory, we propose an updated version of SPEAK-12 for the prediction of the probability of good language outcome at 6 months: SPEAK-6. Further external validation of SPEAK-12 and SPEAK-6 is recommended. Special attention should be given to timing, as time after stroke onset at which predictors and outcome data are collected appears crucial for adequate model validation. Our results show that SPEAK-6 may be used in daily practice to discriminate between stroke patients with good and patients with poor language outcome at 6 months after stroke.

## Additional file

Additional file 1: **Appendix 1.** The SPEAK-model. (DOCX 40 kb)

## Abbreviations

ASRS: Aphasia Severity Rating Scale
AUC: Area Under the ROC Curve
RATS-3: Rotterdam Aphasia Therapy Study – 3
RCT: Randomized Controlled Trial
ROC: Receiver Operation Characteristic
SPEAK: Sequential Prognostic Evaluation of Aphasia after stroKe
