# Supplementary material for: Validation of a prediction model for long-term outcome of aphasia after stroke
Source: BMC Neurol. 2018 Oct 15;18:170. doi: 10.1186/s12883-018-1174-5 (PMC6191997; doi:10.1186/s12883-018-1174-5)
Supplement: Supplementary file 1 — Appendix 1. The SPEAK-model. (DOCX 40 kb) [file 12883_2018_1174_MOESM1_ESM.docx]

**Additional file 1: Appendix 1.** The SPEAK-model.

Box A1 shows the prognostic model by El Hachioui et al. (2013) predicting the probability of good outcome from aphasia one year after stroke.

Box A1. The SPEAK-model, SPEAK-12.

***P* (ASRS = 4 or 5) = e^y^ / (1 + e^y^)**

y = 2.04 + 0.27(Phonology score) + 0.10(Barthel score) – 0.06(age) – 0.76(education level) + 0.27(cardio-embolic infarction) + 2.18(intracerebral hemorrhage)

e = 2.718 (constant)

Variables:

- Phonology Score: score on ScreeLing subpart *Phonology* (score range: 0-24)
- Barthel score: score on *Barthel Index* (score range: 0-20)
- Age: age at stroke
- Educational level: high = 0 (junior high school or middle vocational education up to university), low = 1 (unfinished elementary school up to sophomore high school or lower vocational education)
- Cardio-embolic infarction: yes = 1, no = 0
- Intracerebral hemorrhage: yes = 1, no = 0

Box A2 shows the updated SPEAK-model, SPEAK-6, based on RATS-3 data predicting the probability of good outcome from aphasia six months after stroke.

Box A2. The updated SPEAK-model, SPEAK-6.

***P* (ASRS = 4 or 5) = e^y^ / (1 + e^y^)**

y = 0.06 + 0.27(Phonology score) + 0.10(Barthel score) – 0.06(age) – 0.76(education level) + 0.27(cardio-embolic infarction) + 2.18(intracerebral hemorrhage)

e = 2.718 (constant)

Variables:

- Phonology Score: score on ScreeLing subpart *Phonology* (score range: 0-24)
- Barthel score: score on *Barthel Index* (score range: 0-20)
- Age: age at stroke
- Educational level: high = 0 (junior high school or middle vocational education up to university), low = 1 (unfinished elementary school up to sophomore high school or lower vocational education)
- Cardio-embolic infarction: yes = 1, no = 0
- Intracerebral hemorrhage: yes = 1, no = 0
